# Voltammetric Determination of Glutathionein Pharmaceutical and Biological Samples Using Multiwall Carbon Nanotubes Paste Electrode in the Presence of Rutin as a Mediator

**DOI:** 10.22037/ijpr.2016.1947

**Published:** 2020

**Authors:** Mohsen Keyvanfard, Zeynab Jalilian, Hassan Karimi-Maleh, Khadijeh Alizad

**Affiliations:** a *Department of Chemistry, Majlesi Branch, Islamic Azad University, Isfahan, Iran. *; b *Department of Chemistry, Shahreza Branch, Islamic Azad University, Isfahan, Iran. *; c *Department of Chemical Engineering, Laboratory of Nanotechnology, Quchan University of Technology, Quchan, Islamic Republic of Iran.*

**Keywords:** Glutathione, Rutin, Multiwall carbon nanotubes, Modified electrode, Voltammetry

## Abstract

A new sensitive and selective electrochemical sensor was developed for electrocatalytic determination of glutathione (GSH) in pharmaceutical and biological samples. GSH is a tripeptidethiol present in all eukaryotic and probiotic cells. A voltammetric study of GSH has been carried out at the surface of carbon paste electrode modified with multiwall carbon nanotubes in the presence of rutin as a mediator. The electrochemical oxidation of GSH was investigated by cyclic voltammetry, chronoamperometry and square wave voltammetry (SWV) techniques. Under the optimized conditions, the peak current was linear to GSH concentration over the concentration range of 0.3 to 180μmol L^−1^ using SWV. The detection limit was 0.09μmol L^−1^. The proposed method was successfully applied to the determination of GSH in the urine, tablet and hemolysed erythrocyte samples.

## Introduction

Electrochemical sensing based on carbon nanotubes (CNTs) is now a developed research field. Several advantages of CNTs as electrode materials have been attested for analysis of diversified chemicals of food quality, pharmaceutical and environmental interest (1-3). CNT-modified sensors exhibit low limit of detection (LOD) and fast response due to the signal enhancement provided by high surface area, low overvoltage, and rapid electrode kinetics (4,5). A thermal conductive, mechanically strong and chemically stable nature of CNTs is very appealing for sensing applications (6-8). Their high surface-to-volume ratio of nano-materials and especially carbon nanotubes is also a definite asset toward the development of electrochemical platforms for electro-active compounds detection (9-15).

Drug analysis plays important roles in drug quality control, and has a great impact on public health (16-21). Therefore, a simple, sensitive and accurate method for the determination of active ingredient is very important.

Biological thiols, such as glutathione (GSH) occur widely in living tissues. GSH is the most abundant low molecular mass thiol found in cells. GSH plays a vital role inhuman metabolism, including the detoxi¢cation of xenobiotics, cell homeostasis, radioprotection and antioxidant defense (22). Newly, it has been reported that GSH is incorporated into certain proteins in response to oxidative stress and may participate in posttranslational protein modication (23). So, determination of this compound is important in biological and pharmaceutical samples. The objective of this research is to develop a novel, sensitive, selective and simple electrochemical method with good reproducibility and repeatability for the determination of GSH using unique properties of MWCNTs as a sensor and rutin as a mediator.

## Experimental

*Apparatus and reagents*

All the voltammetric measurements were performed using an AutolabPGSTAT 302N, potentiostat/galvanostat (Utrecht, The Netherlands) connected to a three-electrode cell, Metrohm (Herisau, Switzerland) Model 663 VA stand, linked with a computer (Pentium IV, 1,200 MHz) and with Autolab software. A platinum wire was used as the auxiliary electrode. MWCNTPE and Ag/AgCl/KCl_sat_ were used as the working and reference electrodes, respectively. The electrode prepared with carbon nanotubes was characterized by scanning electron microscopy (SEM) (Seron Tech. AIS 2100). A digital pH/mV-meter (Metrohm model 710) was applied for pH measurements. Spectrally pure graphite powder (particle size < 50 µM) from Merck and multiwall carbon nanotubes (> 90% MWCNTs basis, d × l = (110–70 nm) × (5**–**9 μM) from Fluka were used as the substrate for the preparation of the carbon paste electrode.

*Preparation of the electrode*

Graphite powder (0.900 g) was dissolved in diethyl ether and hand mixed with 0.100 g carbon nanotubes in a mortar and pestle. The solvent was evaporated by stirring. A syringe was used to add paraffin to the mixture, which was mixed well for 40 min until a uniformly wetted paste, was obtained. The paste was then packed into a glass tube. Electrical contact was made by pushing a copper wire down the glass tube into the back of the mixture. When necessary, a new surface was obtained by pushing an excess of the paste out of the tube and polishing it on a weighing paper.

*Preparation of real samples*

For preparation of tablet solution, five tablets of glutathione, labelled 100mg per tablet (Chongqing Yaoyou Pharmaceutical Co., Ltd.). Then, 10mg of each tablet powder was accurately weighed and dissolved in 100mL water by ultrasonication. After mixing completely, the mixture was filtered on an ordinary filter paper, 10 mL of which was subsequently transferred into a 100-mL volumetric flask and diluted to the mark with water. Then, 1.0mL of the solution plus 4.5ml of the buffer (pH 5.0) was used for analysis using the standard addition method.

The urine samples were taken from humans and were used for measurements after its centrifuged (2,500 rpm, 25 °C) and diluted two times with water without any further pretreatment. The standard addition method was used for the determination of the GSH contents after dilution of the sample.

Human whole blood samples were obtained from the Isfahan University Health Centre. Erythrocyteserythrocyte was prepared according to reported procedure (24).

*Optimization of rutin concentration*

The influence of rutin concentration on the peak currents was studied in the concentration range of 0.5-3.0mmol L^-1^rutin at pH = 4.0. The results showed that by increasing the rutin concentration up to 1.0mmol L^-1^ the net peak current increased, whereas further increasing the concentration of rutin caused a decrease in the magnitude of the peak current. Therefore, 1.0 mmol L^-1^ was selected as the optimal rutin concentration.

## Results and Discussion

*SEM characterization*

**Figure 1:**
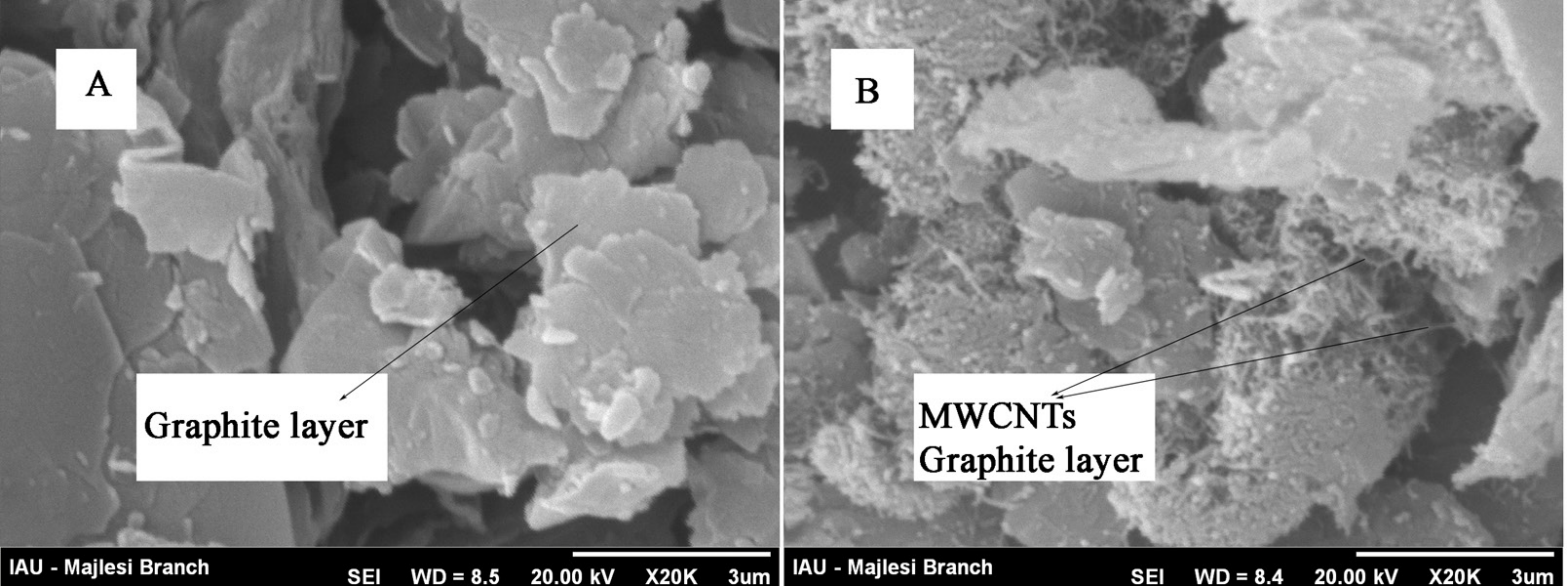
SEM image of a) CPE, b) MWCNTPE

Figure 1 shows SEM images for MWCNTPE and CPE. Result shows, at a surface of CPE (Figure 1A), the layer of irregularly flakes of graphite powder were present and isolated with each other. After multiwall carbon nanotubes (MWCNTs) added to carbon paste matrix, it can be seen that MWCNTs were distributed on the surface of electrode with special three-dimensional structure (Figure 1B), indicating that the MWCNTs were successfully modified on the MWCNTPE.

*Electrochemistry of rutin*

**Figure 2:**
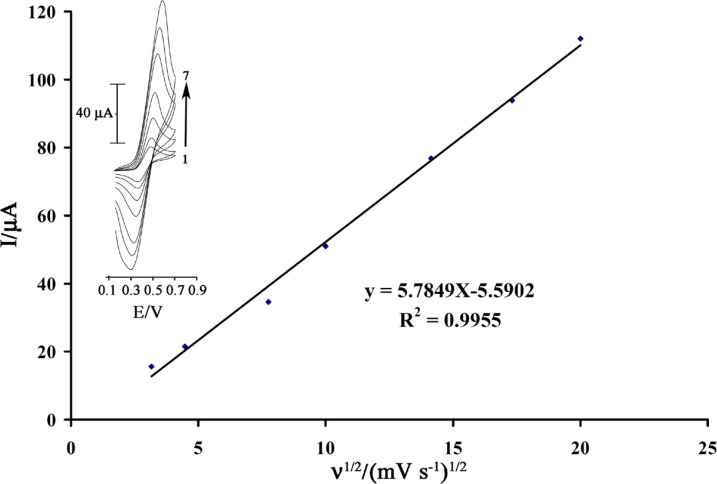
Plot of I_pa_ versus ν^1/2 ^for the oxidation of 1.0 mmol L^-1^rutin at a surface of MWCNTPE. Insert cyclic voltammograms of at various scan rates: (1) 10; (2) 20; (3) 60; (4) 100; (5) 200; (6) 300 and (7) 400 mV s^–1^ in 0.04 universal buffer (pH = 4.0).

The cyclic voltammograms of rutin at a surface of MWCNTPE in 0.04mol L^−1^universal buffer (pH = 4.0) are shown in Figure 2. In set. As can be seen, the cyclic voltammogram exhibits an anodic peak at the forward scan of the potential related to the oxidation of the rutine_(red)_ to rutin_(ox)_. In the reverse scan of the potential, a cathodic peak appears related to the reduction of rutin_(ox)_ to rutine_(red)_. A pair of quasi-reversible peaks are observed at E_pa_ = 0.50V and E_pc_ = 0.33V vs.Ag/AgCl. The half-wave potential (E_1/2_) was 0.43V vs. Ag/AgCl and ΔE_p_ (E_pa_−E_pc_) was 0.17 V. The electrode process was quasi-reversible, with ΔE_p_, greater than the expected value (59/nmV) fora reversible system. The plot of the anodic peak current was linearly dependent on ν^1/2^ for all scan rates (Figure 2). This behavior indicates that the nature of the redox process is diffusion controlled.

The active surface areas of the modified electrodes are estimated according to the slope of the I_p_ vs. ν^1/2^ plot for a known concentration of K_2_Fe (CN)_6_, based on the Randles–Sevcik equation (25):

I_p_ = 2.69 × 105n^3/2^AD_R_^1/2 ^ν^1/2^C_0_      (1)

Where I_pa_ refers to the anodic peak current, n the electron transfer number, A the surface area of the electrode, D_R_ the diffusion coefficient, C_0_ the concentration of K_2_Fe(CN)_6_ and ν is the scan rate. For1.0mmolL^−1^K_2_Fe (CN)_6_ in 0.10 mol L^−1^KCl electrolyte with n=1and D_R_ = 7.6×10^−6^ cms^−1^ and from the slope of the I_pa_–ν^1/2 ^relation, the microscopic areas were calculated. The active surface areas were equal to 0.05 and 0.091 cm^2^ for CPE, MWCNTPE. The result shows that thepresences of MWCNTPE cause increasing the active surface of the electrode.

*Electrocatalytic investigation of GSH*

**Figure 3:**
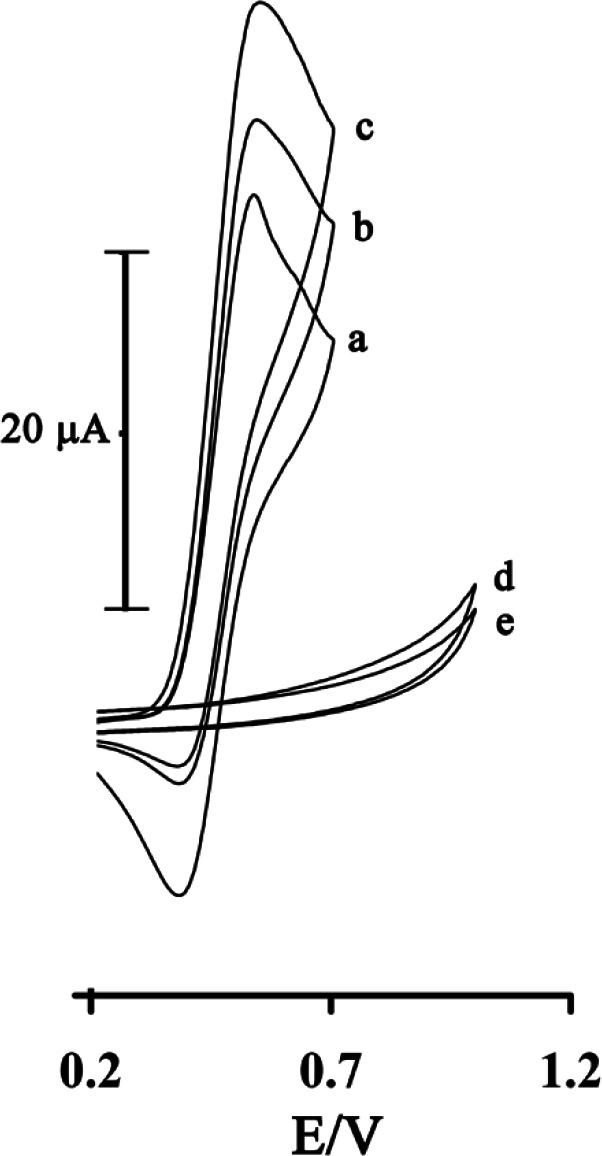
Cyclic voltammograms of 1.0 mmol L^-1^rutinat the surface of MWCNTPE in 0.04 µmol L^-1^universal buffer solution (pH = 4.0) at a scan rate of 20 mV s^−1^ in the absence (a) and in the presence of 100 µmol L^-1^GSH (c). (b) as (c) for the carbon paste electrode. (d) as (c) and (e) as (b) in the absence of rutin

Figure 3 shows the electrocatalytic oxidation of GSH in the presence of rutin at a MWCNTPE surface. As is obvious, at the potential range studied (0.2–1.0 V), GSH was not electroactive at a surface of MWCNTPE and CPE (Figure 3(d and e)), respectively.

On the other hand, the anodic current of rutin was increased substantially in the presence of low concentrations of GSH at a surface of MWCNTPE and CPE (Figure3(c) and 3b)), respectively. This observation is an evidence for electrocatalytic oxidation of GSH by rutin. Similarly, when we compared the oxidation of GSH at the surface of MWCNTPE (curve c) and at CPE (curve b) in the presence of mediator, a dramatic enhancement was observed in the anodic peak current at MWCNTPE vs. the value obtained with CPE. In other words, the data obtained clearly showthat the combination of MWNTs and the mediator definitely improve the characteristics of the electrode for the oxidation of GSH.

**Figure 4:**
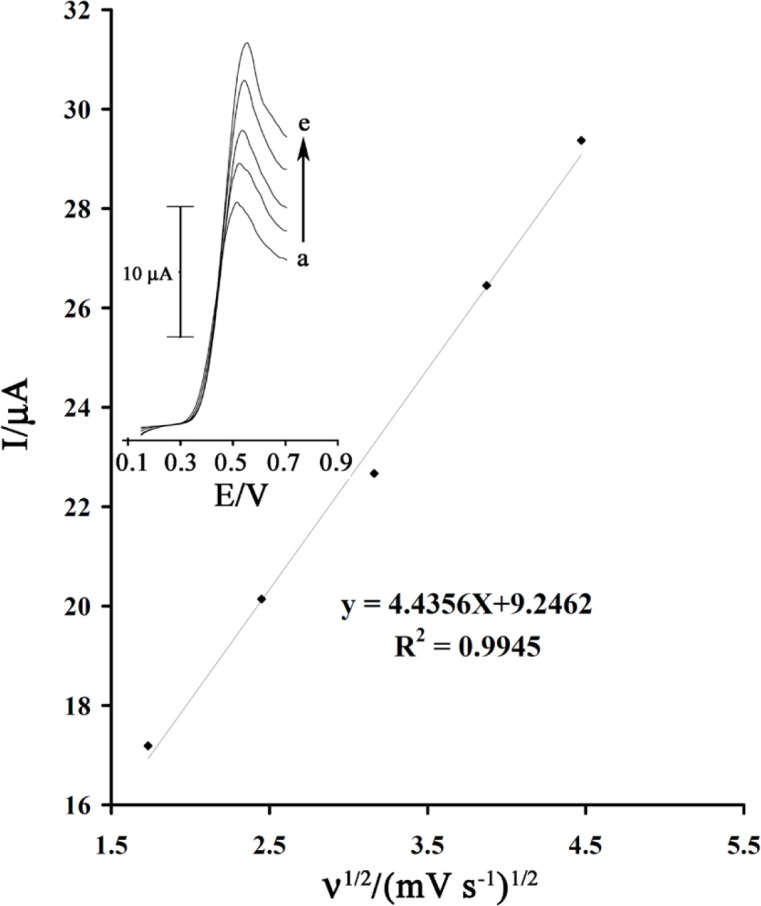
Plot of I_pa_ versus ν^1/2^ for the oxidation of 80 µmol L^-1^GSH in the presence 1.0mmol L^-1^rutin at the surface of MWCNTPE. Inset) Linear sweep voltammograms of 80 µmol L^-1^GSH in the presence 1.0mmol L^-1^rutinat various scan rates as 1) 3, 2) 6, 3) 10, 4) 15 and 5) 20 mV s^−1^ in 0.04 µmol L^-1^ buffer solution (pH = 4.0)

The scan rate dependence of linear sweep voltammograms of a GSH solution (80 µmol L^-1^) in the presence of rutin (1.0 mM) was studied (Figure 4 inset) at a MWCNTPE surface. Figure 4 shows that the anodic peak current increases linearly with the square root of the sweep rate as expected for a diffusion controlled reaction. In addition, with increasing potential scan rate, the catalytic oxidation peak potential gradually shifts towards more positive potentials, suggesting a kinetic limitation in the reaction between rutin and GSH.

To find further information on the rate determining step, a Tafel plot was developed for the MWCNTPE in the presence of mediator using the data derived from the raising part of the current–voltage curve. The slope of the Tafel plot is equal to n (1−α) F/2.3RT which comes up to 9.5010 Vdecade^−1^. We obtained n_α_ as 0.44. Assuming n = 1, then α= 0.44.

*Influence of pH*

In order to optimize the electrocatalytic response of the sensor to GSH oxidation, we investigated the effect of solution pH on the electrocatalytic oxidation of GSH in 0.04 mol L^–1^universal buffer solutions with different pH values (2.0<Ph <6.0) using rutin as mediator at a surface of MWCNTPE. The influence of pH on both peaks current and peaks potential were assessed by examining the electrode responses in the buffer solutions. The results show that maximum electrocatalytic current was obtained at pH 4.0. Therefore, a pH value of 4.0 was chosen as the optimum value for the determination of GSH at MWCNTPE in the presence of rutin.

*Chronoamperometric studies*

**Figure 5:**
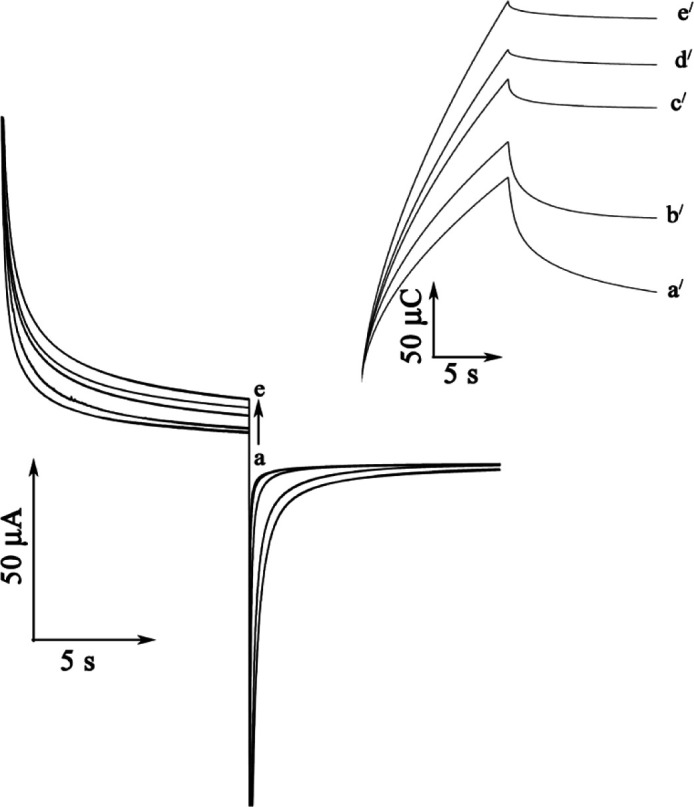
**A)** Chronoamperograms obtained at the MWCNTPE in the absence a) and in the presence of b) 350; c) 450; d) 500 and e) 600 µmol L^-1^ GSH in a buffer solution (pH = 4.0). B) The charge-time curves a') for curve (a); b') for curve (b); c^/^) for curve (c); d^/^) for curve (d) and e^/^) for curve e

In order to obtain an estimation of the rate constant of the catalytic oxidation (k^/^_h_) of GSH, chronoamperometric method was applied to the system (Figure 5A). The rate constant for the chemical reaction between rutin and GSH (k_h_) is determined according to the method of Galus (26).

I_C_/I_L_ = π^1/2^ γ^1/2^ = π^1/2^(k_h_t)^1/2^     (2)

where I_C_ is the catalytic current of rutin in the presence of GSH and I_L_ is the limiting current in the absence of GSH. From the slope of I_C_/I_L_ versus t^1/2^ for five different concentrations of GSH, the average value of k_h_ was calculated to be 8.81 × 10^2^M^−1^ sec^−1^ (Not shown). This value of rate constant explains the sharp catalytic peak observed for the oxidation of GSH at the surface of MWCNTPE in the presence of mediator.

Figure 5B shows the double-potential step chronocolougrams for the mediator in the absence and presence of different concentration of GSH at a surface of MWCNTPE. The results show that forward and backward potential step chronocoloumetry in a blank buffer solution yields very symmetrical chronocolougrams. These had about an equal charge consumed for both oxidation and reduction of the redox system in the mediator at a surface of MWCNTPE. However, in the presence of GSH, the charge value associated with forward chronocoloumetry was significantly greater than that observed for backward chronocoloumetry. This behavior is typically expected for electrocatalysis at chemically modified electrodes (17-32).

*Interference studies*

Interference studies were carried out with several chemical substances prior to the application of the proposed method for the assay of GSH in hemolysed erythrocyte, urine and tablet. The potential interfering substances were chosen from the group of substances commonly found with GSH in pharmaceuticals and in biological fluids. The influence of various substances as potential interference compounds on the determination of 5.0 μmol L^-1^ GSH under the optimum conditions was studied. Tolerance limit was defined as the maximum concentration of the interfering substance that caused an error less than 5% for determination of GSH. The results are given in Table 1 which shows the peak current of GSH is not affected by all conventional cations, anions, and organic substances.

*Dynamic range and limit of detection*

Square wave voltammetry (SWV) was used to determine the concentration of GSH. The square wave voltammograms clearly showed two linear dynamic ranges that the plot of the peaks current versus GSH concentrations were linear. For 0.3–17µmol L^-1^ GSH, the regression equation was I_p_(µA)=(0.085±0.003)C_GSH_+(2.806±0.068) (r^2^=0.992, n = 5) and for 17–180µmolL^-1^GSH, the regression equation was Ip(µA)=(0.008±0.001)C_GSH_+(4.019±0.513) (r^2^=0.9947, n = 8). The detection limit (3σ) was 0.09μmol L^-1^.

The repeatability and stability of the MWCNTPE in the presence of rutin were investigated by cyclic voltammetry measurements of 5.0 µmol L^−1^GSH. The relative standard deviation (RSD%) for six successive assays was 1.2%. When using five different electrodes, the RSD% for four measurements was 1.9%. When the electrode was stored in our laboratory at room temperature, the modified electrode retained 97%of its initial response after a week and 95% after 30 days. These results indicate that MWCNTPE in the presence of mediator has both a good stability and a satisfactory reproducibility so that it can be used for GSH determination.

*Determination of GSH in real samples*

In order to evaluate the applicability of the modified electrode for measuring GSH in real samples, GSH values in human erythrocyte, tablet, and urine samples were determined using the proposed method. In addition, the results were compared with those obtained from the spectrophotometric method (33) which is usually used as the standard method for GSH determination. The results are reported in Table 2.

**Table 1:** Interference study for the determination of 5.0 µmol L^-1^ GSH under the optimized conditions

**Species**	**Tolerance limits (W/W)**
Li^+^, Cl^-^, NO_3_^-^ , Hystidine, Alanine, Phenyl alanine, Methionine, Glycine, Methanol, Ethanol, SCN^-^,SO_4_^2-^, Br^-^, L-Theronine,L-isoleucin, Glucose , Fructose, Lactose , Sucrose, Urea; L_Orinthime, Ca^+2^, Mg^+2^	1000
Starch	Saturation
Ascorbic acid	5

**Table 2:** Concentration values obtained from the proposed and Elman methods for GSH analysis in hemolysed erythrocyte, urine and tablet

t_tab (98%)_	**t**_ex_	**F**_tab, (0.05);2,2_	**F**_ex_	**Elman method** **(m** **mol L** ^–1^ **)**	**Proposed method** **(m** **mol L** ^–1^ **)**	**Sample**
3.8	1.8	19	4.5	4.48± 0.05	4.45± 0.03	1.Hemolysed erythrocyte
3.8	3.3	19	8.5	3.49 ± 0.10	3.65± 0.08	2
3.8	2.4	19	6.3	5.37 ± 0.08	5.48 ± 0.05	3
3.8	2.0	19	5.5	5.98 ± 0.06	6.01 ± 0.06	4
–	–	–	–	<LOD	<LOD	5Urine
3.8	2.2	19	6.5	15.37 ± 0.41	15.22 ± 0.41	6
3.8	2.9	19	7.3	30.67 ± 0.72	30.44 ± 0.50	7
3.8	1.0	19	4.3	5.22± 0.33	5.11 ± 0.21	8 Tablet
3.8	1.5	19	5.2	9.88 ± 0.41	10.23 ± 0.30	9

F_ex_ Calculated F-value; Reported F value from F-test table with 95% confidence level and 2/2 degree of freedom; t_ex_Calculated t; t_tab_ (98%) Reported t value from t-student test table with 98% confidence level.

## Conclusions

In this study, rutin was used as a mediator for the homogeneous electrocatalytic oxidation of GSH in aqueous media (pH = 4.0) at the surface of a MWCNTPE. The electrochemical characteristics of rutin and its catalytic effect on the oxidation of GSH were investigated. The rate constant of the catalytic reaction was estimated using chronoamperometry. Square wave voltammetry was successfully applied to the determination of GSH in the presence of an optimum concentration of mediator. Finally, this sensor was used for the determination of GSH in biological and pharmaceutical real samples such as the hemolysed erythrocyte and tablet using standard addition method.
